# Paper Notebooks vs. Mobile Devices: Brain Activation Differences During Memory Retrieval

**DOI:** 10.3389/fnbeh.2021.634158

**Published:** 2021-03-19

**Authors:** Keita Umejima, Takuya Ibaraki, Takahiro Yamazaki, Kuniyoshi L. Sakai

**Affiliations:** ^1^Department of Basic Science, Graduate School of Arts and Sciences, The University of Tokyo, Tokyo, Japan; ^2^NTT Data Institute of Management Consulting, Inc., Tokyo, Japan

**Keywords:** memory encoding, memory retrieval, hippocampus, language, fMRI

## Abstract

It remains to be determined how different inputs for memory-encoding, such as the use of paper notebooks or mobile devices, affect retrieval processes. We compared three groups of participants who read dialogues on personal schedules and wrote down the scheduled appointments on a calendar using a paper notebook (Note), an electronic tablet (Tablet), or a smartphone (Phone). After the retention period for an hour including an interference task, we tested recognition memory of those appointments with visually presented questions in a retrieval task, while scanned with functional magnetic resonance imaging. We obtained three major results. First, the duration of writing down schedules was significantly shorter for the Note group than the Tablet and Phone groups, and accuracy was much higher for the Note group in easier (i.e., more straightforward) questions. Because the input methods were equated as much as possible between the Note and Tablet groups, these results indicate that the cognitive processes for the Note group were deeper and more solid. Second, brain activations for all participants during the retrieval phase were localized in the bilateral hippocampus, precuneus, visual cortices, and language-related frontal regions, confirming the involvement of verbalized memory retrieval processes for appointments. Third, activations in these regions were significantly higher for the Note group than those for the Tablet and Phone groups. These enhanced activations for the Note group could not be explained by general cognitive loads or task difficulty, because overall task performances were similar among the groups. The significant superiority in both accuracy and activations for the Note group suggested that the use of a paper notebook promoted the acquisition of rich encoding information and/or spatial information of real papers and that this information could be utilized as effective retrieval clues, leading to higher activations in these specific regions.

## Introduction

The properties of human memory have been investigated with several approaches, including clinical, psychological, and neuroimaging studies (Tulving, 2002; Schacter et al., 2007; Miyashita, 2019). It remains to be elucidated how brain activations during retrieval processes are modulated by different encoding procedures, because it has been reported that retrieval performances on paired words became worse when the categorically similar target words were simultaneously encoded, suggesting the importance of the context-dependent encoding (Nairne, 2002; Goh and Lu, 2012). It is also possible that the manner with which specific information is encoded—e.g., whether by using a paper notebook, computer, or mobile device—may affect retrieval processes. A recent behavioral study showed that students who took longhand notes performed better on conceptual questions than those who took notes on laptop computers (Mueller and Oppenheimer, 2014). A reasonable explanation for this interesting finding would be that the use of a paper notebook enables users to summarize and reframe information in their own words for encoding, while the use of a laptop tends to encourage them to write down information more passively (i.e., more nearly verbatim). The former processes thus naturally ensure deeper and more solid encoding *via* the active process of making notes. Moreover, it has been reported that longhand note-taking enhanced the performance of students on recognition of memorized words, even though typing on a computer keyboard allowed greater speed (Aragón-Mendizábal et al., 2016).

Another possible explanation for the superiority of longhand note-taking for conceptual understanding is related to the use of paper for writing/reading since a behavioral study reported the superiority of paper to computer screens in terms of reading comprehension (Wästlund et al., 2005; Mangen et al., 2013). These studies indicated the importance of visual and tactile cues for perceiving *constant* physical sizes and spatial locations, because “the material substrate of paper provides physical, tactile, spatiotemporally fixed cues to the length of the text” (Mangen et al., 2013). We hypothesized that the use of a paper notebook, together with longhand note-taking, would enhance both memory encoding and later retrieval processes that could then be investigated at the brain level. More specifically, the utilization of the paper likely enhances the processes of associating episodic (*what*) and spatial (*where*) information, especially in the hippocampus, given its well-established role in the integration of what/where/when information (Broadbent et al., 2004; Eichenbaum, 2004; Chadwick et al., 2010).

To address this issue, we compared three groups of participants who used a paper notebook (Note), electronic tablet (Tablet), or smartphone (Phone) during the encoding phase. Participants in the Tablet group used a stylus pen, thereby controlling for the effects of longhand writing with a pen in the Note group. It should be noted that physical sizes and spatial locations of a document remain constant for a paper notebook, whereas they become variable on the display of a tablet or smartphone. Moreover, not only the physical interaction of the hand with the pen/paper during note-taking but the actual writing of notes relative to each page of the real paper provides more concrete encoding information, because that information can be easily erased and updated by new information on the physically same screen of a tablet or smartphone.

**Figure 1:**
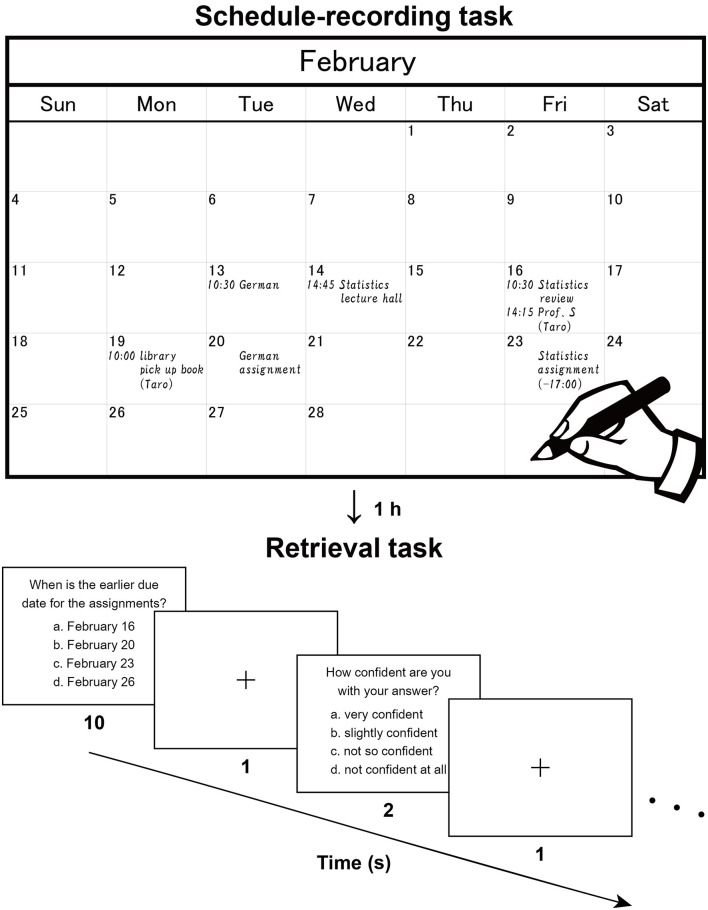
Recording and retrieval of schedule information. Participants first read dialogues (in Japanese), then extracted scheduled appointments contained in the dialogues, and wrote them down with a paper notebook (*Note* group), electronic tablet (*Tablet* group), or smartphone (*Phone* group). This procedure reproduces the daily making of to-do lists and naturally involves encoding processes. The upper panel shows a typical example (English translation) written by a participant. After an hour including an interference task, the participants were asked to answer questions about the appointments and reported their level of confidence in their answer to each question. The lower panel shows a typical trial in this retrieval task.

We asked participants to write down scheduled appointments, and then, after one hour during which they performed an interference task, we conducted a retrieval task in which we tested participants’ recognition memory of those appointments (Figure 1). We further hypothesized that the interaction with physical paper, rather than the mental editing/preparation of the notes or the physical act of handwriting, provides episodic and spatial information of notes relative to each page of real paper, together with visual/tactile information from the paper. These properties and cues of papers could help to retrieve specific information, and thus lead to increased activations in specified brain regions for the Note group, compared with the other groups using mobile devices lacking such processes.

It has been proposed that the hippocampus and the prefrontal cortex support complementary functions in episodic memory and that the bidirectional information flow between these regions may play a crucial role in integrating and consolidating individual information (Moscovitch et al., 2016; Eichenbaum, 2017). A previous functional magnetic resonance imaging (fMRI) study reported that episodic memory of a word or picture is related to a functional network that includes the left posterior precuneus and the left lateral prefrontal cortex (Lundstrom et al., 2003). On the other hand, language function is critically involved in human episodic memory, and some language-related regions would be recruited during both memory encoding and retrieval. The left lateral premotor cortex (LPMC) and left opercular/triangular parts of the inferior frontal gyrus (F3op/F3t) are suggested to have central roles in syntactic processing, whereas the left angular/supramarginal gyri (AG/SMG) make a major contribution to lexical processing (Sakai, 2005). Moreover, the right frontal cortex was identified as a supportive region for syntactic processing (Kinno et al., 2014). Activations in these regions would be observed during memory retrieval because fMRI studies showed that the hippocampus and language-related regions involved in the encoding phase were also activated during the retrieval phase (Rugg et al., 2008). The retrieval task we used critically involved episodic memory of scheduled appointments, and thus activations in these regions would be increased more for the Note group than the other groups.

## Materials and Methods

### Participants

University student volunteers (48 native Japanese speakers, 18 females) aged 18–29 years were openly recruited from multiple sources, including the University of Tokyo and Sophia University, as well as the participant pool of the NTT Data Institute of Management Consulting. The laterality quotient (LQ) was measured according to the Edinburgh inventory (Oldfield, 1971); all participants but one were right-handed, and the exception was both-handed (LQ: −14). As stated above, the participants were divided into three groups: Note, Tablet, and Phone groups (Table 1). These three groups were age- and LQ-matched (Kruskal–Wallis test, *p* > 0.1), as well as gender-matched (Fisher’s exact test for count data, *p* = 0.17). Each participant first answered a questionnaire on their daily use of paper notebooks, electronic tablets, and smartphones for scheduling in an academic or personal context (seven-point scale for each). Based on this result, electronic tablet users were assigned to the Tablet group, and smartphone users (those on the highest scale for smartphone use) were assigned to either the Tablet or Phone group. To estimate short-term memory ability, we used the number-letter sequencing in the Wechsler Adult Intelligence Scale—Fourth Edition (Drozdick et al., 2012), and the maximum length of memorized sequences was not significantly different among the three groups (*p* = 0.4). All participants in the Note group used paper notebooks for daily schedule management, whereas eight and seven participants in the Tablet and Phone groups, respectively, also used paper notebooks for that purpose. To control the experience and accustomedness of using paper notebooks for daily schedule management, these 15 participants (with eight females) were separately designated the *Device* group, which was used in behavioral and activation analyses.

**Table 1:** Basic data on participants.

Experimental groups	Number of participants	Age (year)	LQ	The maximum length of memorized sequences
Paper notebook (Note)	16 (8)	20.8 ± 1.6 (18.7–24.1)	90 ± 10 (71–100)	6.5 ± 1.2 (4–8)
Electronic tablet (Tablet)	16 (3)	20.1 ± 1.0 (18.7–22.4)	88 ± 12 (65–100)	6.4 ± 1.0 (5–8)
Smartphone (Phone)	16 (7)	21.8 ± 2.6 (19.0–27.7)	83 ± 28 (−14–100)	6.9 ± 1.1 (4–8)

*For the number of participants, numbers of females are shown in parentheses. For age, laterality quotient (LQ), and the maximum length of memorized sequences, averaged data (mean ± standard deviation) and their ranges (in parentheses) are shown*.

Before they participated in the study, the nature and possible consequences of the studies were explained to each participant and written informed consent was obtained afterward. None of the participants had a history of neurological or psychological disorders. Approval for the experiments was obtained from the institutional review board of the University of Tokyo, Komaba.

### Stimuli and Tasks

Two sets of written dialogues between two or three persons (a set of dialogues on academic matters and a set on personal matters) were presented to the participants, who were asked to imagine that they were participating in those dialogues. There were seven daily scheduled appointments for the academic context and seven for the personal context (in February and March, respectively). While silently reading the dialogues, participants were asked to enter each of these appointments into a monthly calendar (Figure 1, upper panel). The participants used either a paper notebook [Noritsu NOLTY Notebook (2017), size 20.6 × 17.6 cm^2^ when opened], an electronic tablet [iPad Pro 10.5 inch (2017), screen size 21.4 × 16.1 cm^2^ in landscape orientation], or a smartphone (Google Nexus 5 LG-D821, screen size 6.2 × 10.9 cm^2^ in portrait orientation), where the paper notebook and electronic tablet were similar in physical layout (size and orientation). All three types of calendars had a day, week, and month view, but we used only the month view. In the case of the paper notebook and electronic tablet, appointments could only be viewed and edited individually in the relevant month (i.e., discrete views). In the smartphone, individual weeks could be viewed and edited by swiping continuously (i.e., continuous views). This difference was notable, in that schedule information would be encoded relative to the *spatial configuration* of one month (see Figure 1) for the paper notebook and electronic tablet.

Regarding input methods, a four-color pen was used to write in the paper notebook [the use of color(s) was up to each participant], and a stylus pen was used to write on the electronic tablet with a free choice of multiple colors (without using a virtual keyboard). In the case of the smartphone, the text was written by either flick input with the finger(s) or by using a virtual keyboard. In Japanese, there are three types of characters (*hiragana*, *katakana*, and *kanji*; kanji basically consists of Chinese characters), and kana-kanji transformation is usually used for inputs in mobile devices and computers (kana-kanji transformation converts a limited number of hiragana to vast numbers of kanji by requiring users to select appropriate kanji from multiple candidates). The flick input utilizes a telephone keypad with a three by four layout, and one hiragana character can be selected by either tapping a keypad or flicking from a keypad to one of four directions (up, down, left, or right) to enter one of five hiragana characters sharing the same initial consonant.

We measured the time required by participants to write down the appointments, but we set no time limit. When the participants finished writing down, they were instructed to review the calendar for 30 s. Then, after the retention period for an hour including an interference task, participants were asked to recall those appointments in a retrieval task; the experimental purpose of writing down the appointments was not disclosed to them. The interference task involved listening comprehension; participants were informed that they would hear a story, and then be asked about its contents while lying in an MRI scanner. We used the first 6 min of a narrated version of a Japanese classic short story called “*Ma-jutsu* (*Magic*)” (written by Ryūnosuke Akutagawa, narrated by Takeshi Sasaki, and published by Pan Rolling, Japan). This story was unfamiliar to all participants. The auditory stimuli were presented through a headphone and participants were not permitted to take notes while listening. Sixteen questions about the detailed contents of the story were displayed inside the scanner (two questions per run), and the participants pressed one of four buttons to select the right answer.

After a short break outside the scanner to adjust the time between the encoding and retrieval phases to 1 h, participants performed a retrieval task inside the scanner (Figure 1, lower panel), in which 16 questions about detailed contents of the appointments were displayed (two questions per run). Out of the 16 questions, seven required recalling of the relationships between multiple appointments, one required the conversion from the date to the day of the week (using the spatial information of the calendar), and three required recalling from similar or confusing appointments. The remaining five questions were more straightforward and thus considered as the *easier* questions. In each trial, a question was presented with four choices, and the participants pressed a button to select the right answer within 10 s. After an interval of 1 s, participants reported their level of confidence (1–4 scale, 4 = very confident) for that answer by pressing one of four buttons within 2 s. These responses were used to assess the correctness of each participant’s self-evaluation, where the true positive rate vs. the false positive rate was plotted for each of the four levels of confidence. By connecting these plots, we obtained a receiver operating characteristics curve (Fawcett, 2006), and we used the area under the curve (AUC) for this assessment (0 = perfectly wrong; 0.5 = no distinction; 1 = perfectly correct).

As a control condition, we added a 2-back task into the run with the retrieval task. In each trial of the 2-back task, two different non-words, each with three Japanese characters, were sequentially displayed (each for 2 s). These characters were randomly selected from those used in the retrieval tasks, where the same type of characters (either hiragana, katakana, or kanji) was presented in a block of trials. Then four choices were shown for 5 s with a new non-word to be remembered. The correct answer was the non-word that appeared 2-back before but in a different order of three characters. There were two to four continuous trials with button pressings in each block.

Each run consisted of three 2-back blocks and two retrieval task trials, in which a 2-back block always started first, and the 2-back blocks and retrieval task trials were alternated. As fMRI events, we estimated the 6-s memory retrieval phase [determined by response times (RTs)] and the subsequent 4-s *post hoc* period from each 10-s period of the retrieval task, as well as a 5-s event for the 2-back task. With regards to contrasts between events, we always applied an exclusive mask of negative activations for the control conditions (one-sample *t-test*, uncorrected *p* < 0.05). During the scans, the participants wore earplugs and an eyeglass-like MRI-compatible display (resolution = 800 × 600 pixels, framerate = 60 fps; VisuaStim Digital, Resonance Technology Inc., Northridge, CA, USA). The stimuli were all presented in yellow letters on a black background. For fixation, a small red cross was shown at the center of the screen when a stimulus was not shown. The stimulus presentation and collection of behavioral data (accuracy and RTs) were controlled using the Presentation software package (Neurobehavioral Systems, Albany, CA, USA).

### MRI Data Acquisition

The MRI scans were conducted in a 3.0 T scanner (Signa HDxt; GE Healthcare, Milwaukee, WI, USA) with a bird-cage head coil. Each participant was in a supine position, and his or her head was immobilized inside the coil. As regards the structural images, high-resolution T1-weighted images of the whole brain (136 axial slices, 1 × 1 × 1 mm^3^) were acquired with a three-dimensional fast spoiled gradient-echo (3D FSPGR) acquisition [repetition time (TR) = 8.6 ms, echo time (TE) = 2.6 ms, flip angle (FA) = 25°, field of view (FOV) = 256 × 256 mm^2^]. With respect to the time-series data of fMRI, we used a gradient-echo echo-planar imaging (EPI) sequence (TR = 2 s, TE = 30 ms, FA = 78°, FOV = 192 × 192 mm^2^, resolution = 3 × 3 mm^2^). We scanned a set of 30 axial slices that were 3-mm thick with a 0.5-mm gap, covering the range of −38.5 to 66 mm from the line of the anterior commissure to the posterior commissure (AC-PC). In a single scanning run, we obtained 45 volumes and dropped the initial four volumes from analyses due to MR signal increases.

### fMRI Data Analyses

The fMRI data were analyzed in a standard manner using SPM12 statistical parametric mapping software (Wellcome Trust Center for Neuroimaging^1^; Friston et al., 1994) implemented on MATLAB (Math Works, Natick, MA, USA). The acquisition timing of each slice was corrected using the middle slice (the 15th slice chronologically) as a reference for the functional images. We spatially realigned each volume to the first volume of consecutive runs, and a mean volume was obtained. We set the threshold of head movement during a single run as follows: within a displacement of 2 mm in any of the three directions, and a rotation of 1.4° around any of the three axes. These thresholds were empirically determined in our previous studies (Kinno et al., 2008). If a run included one or several images over this threshold, we replaced the outlying image with an interpolated image, which was the average of the chronologically former and latter ones, and conducted the realignment procedure again. The realigned data were resliced every 3 mm using seventh-degree B-spline interpolation.

Each individual’s structural image was matched with the mean functional image generated during realignment. The resultant structural image was spatially normalized to the standard brain space as defined by the Montreal Neurological Institute (MNI) using the extended version of the unified segmentation algorithm with light regularization; this is a generative model that combines tissue segmentation, bias correction, and spatial normalization in a single model (Ashburner and Friston, 2005). The resultant deformation field was applied to each realigned functional image to be spatially normalized with non-linear transformation. All normalized functional images were then smoothed by using an isotropic Gaussian kernel of 9 mm full-width at half maximum (FWHM). Low-frequency noise was removed by high-pass filtering at 1/128 Hz.

In the first-level analysis (i.e., the fixed-effects analysis within a participant), each participant’s hemodynamic responses were modeled for the following types of events: initial 2-back trials with encoding alone, other 2-back trials, 6-s memory retrieval phase of retrieval trials, and 4-s *post hoc* period of retrieval trials. These event types were separately set for each group. Each event was modeled with the boxcar function overlaid with a hemodynamic response function. To minimize the effects of head movement, the six realignment parameters obtained from preprocessing were included as a nuisance factor in a general linear model.

These modeled responses were then generated in a general linear model for each participant and used for the inter-subject comparison in a second-level analysis (i.e., the random-effects analysis for a group). To examine the activation of the regions in an unbiased manner, we adopted whole-brain analyses. For statistical analyses, a two-way ANOVA (group × event type) with *t*-tests was performed with three nuisance factors (age, gender, and laterality quotient), where the statistical threshold was set to family-wise error (FWE) corrected *p* < 0.05 for the voxel level. For the anatomical identification of activated regions, essentially we used the Anatomical Automatic Labeling (AAL) method^2^ (Tzourio-Mazoyer et al., 2002) and the labeled data as provided by Neuromorphometrics Inc.^3^, under academic subscription. In addition to whole-brain analyses, we adopted analyses of each region of interest (ROI) by using the MarsBaR-toolbox^4^, in which an ROI was taken from a cluster identified by the “retrieval—2-back” contrast for all participants, which were further extracted with an AAL mask of each region.

## Results

### Behavioral Results

**Figure 2:**
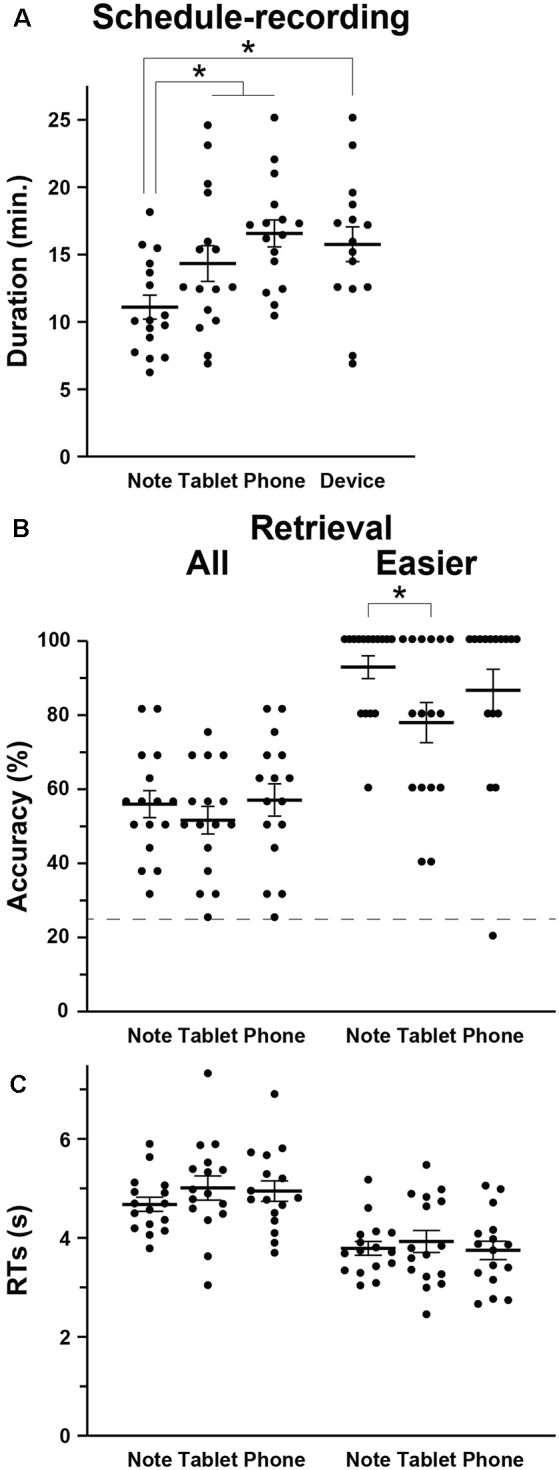
Behavioral data. **(A)** The intergroup differences in the mean duration of schedule recording (see Figure 1), together with individual data points overlapped. In addition to the three groups (Note, Tablet, and Phone), we also introduced a *Device* group, which consisted of participants who used mainly notebooks daily and were assigned to either the Tablet or Phone group. **(B)** Accuracy in the retrieval task. The broken line denotes the chance level of 25% accuracy. For the easier (i.e., more straightforward) questions, the Note group showed significantly higher accuracy than the Tablet group. **(C)** Response times (RTs) in the retrieval task. Error bars indicate standard errors of the mean. *p < 0.05.

We first compared the amounts of time required to write down the scheduled appointments (i.e., the duration of schedule recording) among the Note, Tablet, and Phone groups, and we observed a significant difference by a one-way ANOVA (*F*_(2,45)_ = 6.5, *p* = 0.003; Figure 2A). The duration was significantly shorter for the Note group compared to the Tablet and Phone groups combined (*t-test*, *t*_(46)_ = 3.2, *p* = 0.002). We also confirmed a significant difference between the Note and Device groups (*t*_(29)_ = 3.0, *p* = 0.003).

Relative to the chance level of 25% accuracy, the accuracy for the retrieval task was reliable and well below the ceiling level (Figure 2B). The participants’ self-evaluation on confidence was also correct, because the AUC for the Note, Tablet, and Phone groups were 0.77 ± 0.14, 0.77 ± 0.12, and 0.74 ± 0.11, respectively, where group differences were not significant (*F*_(2,45)_ = 0.2, *p* = 0.8). The accuracy or RTs in the retrieval was not significantly different among the three groups (accuracy: *F*_(2,45)_ = 0.5, *p* = 0.6; RTs: *F*_(2,45)_ = 0.8, *p* = 0.5; Figure 2C); the accuracy and RTs in the interference and 2-back tasks were also comparable among the three groups (*p* > 0.4). However, we observed significant group differences when we focused on the easier questions of scheduled appointments (see “Materials and Methods” section; Figure 2B). According to non-parametric tests for the data showing ceiling effects, the accuracy of the easier questions was significantly higher for the Note group than the Tablet group (Wilcoxon rank-sum test, *W* = 179, *p* = 0.04), and the difference between the Note and Device groups was marginally significant (*W* = 164, *p* = 0.06).

The Tablet and Phone groups (or Device group) took more time for writing down (Figure 2A), and this might be due to slower input of characters with such mobile devices (no typing on the computer keyboard). However, at least between the Note and Tablet groups, the use of a stylus pen was just similar to writing with a four-color pen, and the physical layout of a notebook or tablet was equated as much as possible (see “Materials and Methods” section). Moreover, there was ample time for every group to write down all appointments into a monthly calendar. Therefore, *shorter* amounts of time for writing down and *higher* accuracy in easier questions for the Note group suggest that those cognitive processes for the Note group were actually deeper and more solid.

When all participants in the three groups were combined, the accuracy in the retrieval and 2-back tasks were significantly correlated (Pearson’s correlation, *r* = 0.31, *t*_(46)_ = 2.2, *p* = 0.03). RTs showed a significant correlation as well (*r* = 0.33, *t*_(46)_ = 2.4, *p* = 0.02). These results confirm consistent immediate- and short-term memory capacities for every participant.

### Enhanced Activations in Bilateral Regions for the Note Group

**Figure 3:**
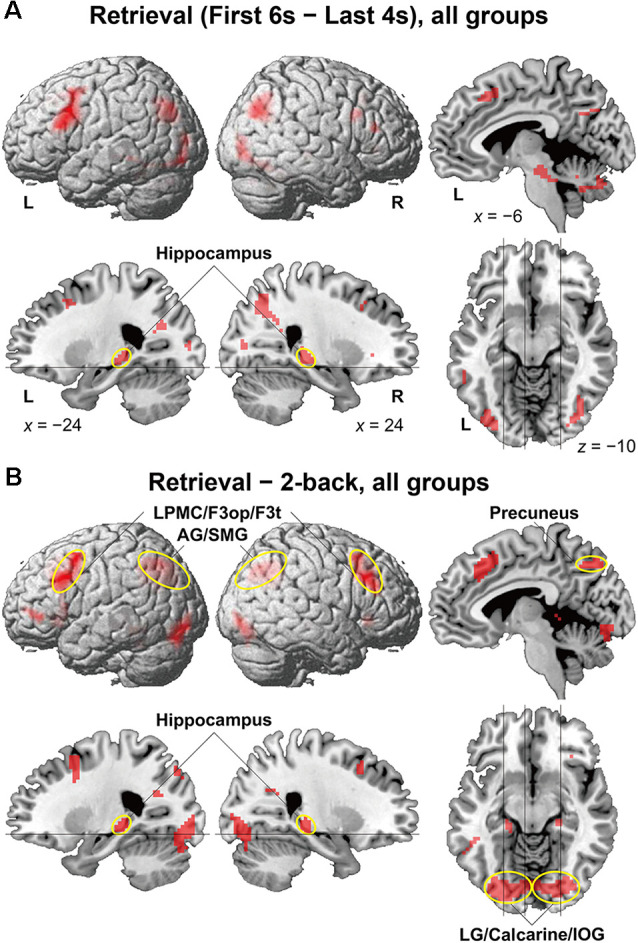
Activated regions for the retrieval task. **(A)** Results of the “First 6 s—Last 4 s” contrast within the retrieval task period are shown for all participants. **(B)** Results of the “retrieval—2-back” contrast are shown for all participants. The lines indicate the locations of the sections. Localized activations were observed bilaterally in the lateral premotor cortex/opercular/triangular parts of the inferior frontal gyrus (LPMC/F3op/F3t), angular/supramarginal gyri (AG/SMG), hippocampus, precuneus, and lingual gyrus/calcarine/inferior occipital gyrus (LG/calcarine/IOG; see Table 2 for the list of local maxima).

To identify brain regions specifically involved in the memory retrieval process, we directly compared activations between the 6-s memory retrieval phase and the 4-s *post hoc* period from each 10-s period of the retrieval task, denoted as “First 6 s—Last 4 s” contrast. This was because the mean RTs were less than 6 s for all but two participants (see Figure 2C). With this stringent contrast during the same stimulus presentation and task, dynamic signal changes induced by such active retrieval processes should be revealed. As shown in Figure 3A, localized activations were found bilaterally in the middle frontal gyrus, F3op/F3t, fusiform gyrus, AG/SMG, middle/inferior occipital gyrus (MOG/IOG), pallidum, and hippocampus; we also observed left-lateralized activation in the LPMC and precuneus.

It is still possible that these activations reflect immediate memory processes that were necessary to solve the retrieval task; note the above-mentioned correlation between performances of the two tasks. Thus, we further compared activations in the retrieval task (10-s period) against those in the 2-back task with more demanding immediate memory, which successfully removed common factors in both tasks (Figure 3B). The result of activations replicated the above-mentioned regions (Table 2), providing appropriate ROIs for further analyses. Additional activations were found bilaterally in the lingual gyrus (LG) and calcarine sulcus; we also observed left-lateralized activation in the orbital part of the inferior frontal gyrus (F3O).

**Table 2:** ROIs determined by the contrast of “retrieval—2-back” for all participants.

Brain regions	BA	Side	*x*	*y*	*z*	*Z*	Voxels
LPMC	6/8/9	L	–36	8	47	Inf	1,030
		R	39	17	53	7.1	*
F3op/F3t	44/45	L	–48	20	8	5.9	*
		R	48	29	35	7.5	*
ACC/pre-SMA	32/8	M	–6	29	44	Inf	*
F3t/F3O	45/47	L	–45	41	–4	7.8	71
Insula	13	L	–30	26	–4	7.0	46
		R	33	29	–4	6.8	49
ITG	20	L	–54	–43	–13	6.6	15
FG	37	L	–36	–46	–22	5.4	12
AG/SMG	39/40	L	–33	–70	35	Inf	246
		R	39	–67	38	Inf	180
Precuneus	7	L	–9	–64	41	7.8	97
			–21	–61	26	6.9	*
		R	24	–58	26	6.0	8
LG/Calcarine/IOG	18/19/17	L	–12	–88	–10	Inf	622
		R	9	–85	–10	Inf	*
Cerebellum Crus I/Crus II/VI		R	12	–79	–28	6.2	*
*ibid*. IV/V		M	–6	–40	–1	5.4	71
Hippocampus		L	–24	–31	–4	7.0	*
		R	24	–28	–4	6.4	31

*Stereotactic coordinates (*x*, *y*, *z*) in the MNI space are shown for activation peaks of Z values which were more than 12 mm apart in either direction of the *x*, *y*, or *z-*axis. FWE corrected *p* < 0.05 for the voxel level. The region with an asterisk is included within the same cluster shown in the nearest row above. BA, Brodmann’s area; L, left; M, medial; R, right; ACC, anterior cingulate cortex; AG, angular gyrus; F3O, orbital part of the inferior frontal gyrus (F3); F3op, opercular part of the F3; F3t, triangular part of the F3; FG, fusiform gyrus; IOG, inferior occipital gyrus; ITG, inferior temporal gyrus; LG, lingual gyrus; LPMC, lateral premotor cortex; MOG, middle occipital gyrus; pre-SMA, pre-supplementary motor area; SMG, supramarginal gyrus*.

**Figure 4:**
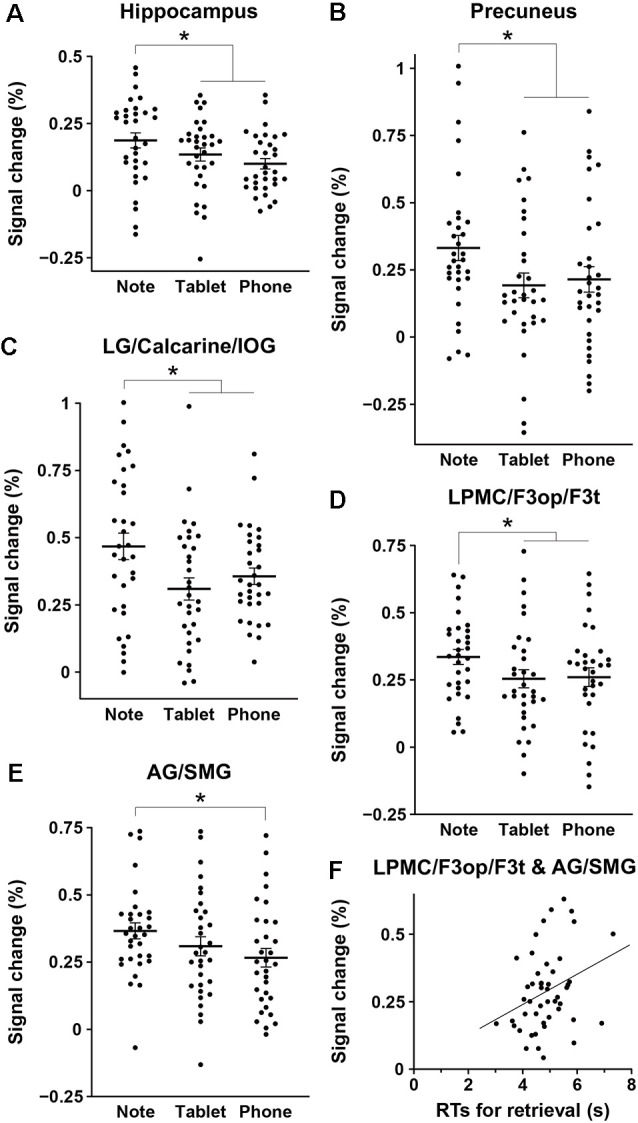
Intergroup differences in brain activations for the retrieval task. **(A–E)** Mean percent signal changes, together with individual data points overlapped, for the three groups in the regions of interest (ROIs) of the hippocampus **(A)**, precuneus **(B)**, LG/calcarine/IOG **(C)**, LPMC/F3op/F3t **(D)**, and AG/SMG **(E)**. The signal changes of an ROI in each hemisphere were treated as independent samples, in reference to those in the 2-back task. Error bars indicate standard errors of the mean. *p < 0.05. **(F)** A significant correlation between the RTs in the retrieval task and the averaged signal changes in the ROIs of the LPMC/F3op/F3t and AG/SMG (the language-related regions) for all participants.

We assessed percent signal changes for these ROIs, and found significant intergroup differences in the posterior hippocampus, precuneus, LG/calcarine/IOG, LPMC/F3op/F3t, and AG/SMG (Figures 4A–E). Activations in the first four regions were significantly different between the Note group and the combined Tablet and Phone groups (hippocampus: *t*_(94)_ = 2.4, *p* = 0.02; precuneus: *t*_(94)_ = 2.3, *p* = 0.03; LG/calcarine/IOG: *t*_(94)_ = 2.7, *p* = 0.008; LPMC/F3op/F3t: *t*_(94)_ = 2.0, *p* = 0.05), whereas those in the last region were significantly different between the Note and Phone groups (*t*_(62)_ = 2.2, *p* = 0.03). Activations in the LG/calcarine/IOG and LPMC/F3op/F3t were also significantly different between the Note and Device groups (LG/calcarine/IOG: *t*_(60)_ = 2.2, *p* = 0.03; LPMC/F3op/F3t: *t*_(60)_ = 2.4, *p* = 0.02), even when the experience/accustomedness of using paper notebooks was equated. Moreover, we observed a significant positive correlation between the RTs in the retrieval task and the averaged signal changes in the ROIs of LPMC/F3op/F3t and AG/SMG for all participants (*r* = 0.31, *t*_(46)_ = 2.2, *p* = 0.03; Figure 4F). This link between behavioral results and brain activations indicates that inner language processes were indeed involved in during memory retrieval *via* the function of the language-related regions.

## Discussion

Using three groups of participants who performed a schedule-recording task using a paper notebook, electronic tablet, or smartphone, followed by a retrieval task (Figure 1), we obtained three major results. First, the duration of schedule recording was significantly shorter for the Note group than the Tablet and Phone groups, and accuracy was much higher for the Note group in easier (i.e., more straightforward) questions (Figure 2). Because the input methods were equated as much as possible between the Note and Tablet groups, these results indicate that the cognitive processes for the Note group were actually deeper and more solid. Second, brain activations for all groups during the retrieval phase were localized in the bilateral hippocampus, precuneus, LG/calcarine/IOG, and LPMC/F3op/F3t (Figure 3), confirming the involvement of verbalized memory retrieval processes for appointments. Third, activations in these regions were significantly higher for the Note group than those for the Tablet and Phone groups (Figure 4). These enhanced activations for the Note group could not be explained by general cognitive loads or task difficulty, because overall task performances were similar among the groups. Brain activations for the Tablet and Phone groups were similar, where the difference in input methods did not affect the results. On the other hand, the Note and Tablet groups showed a clear difference in brain activations even if the physical layout and input methods were controlled. Brain activations were significantly different also between the Note and Device groups, even when accustomedness to paper notebooks or mobile devices was equated for daily usage. The significant superiority in both accuracy and activations for the Note group suggested that the use of a paper notebook promoted the acquisition of rich encoding information and/or spatial information of real papers (see the “Introduction” section) and that this information could be utilized as effective retrieval clues, leading to higher activations in these specific regions.

The hippocampus is crucially involved not only in memory encoding and retrieval processes but also in spatial memory itself. The hippocampal-entorhinal cortex provides spatial representations, as demonstrated by grid cells (Hartley et al., 2014; Moser et al., 2015). It has also been suggested that activations in the human hippocampus encode distances between locations in the real world (Morgan et al., 2011; Howard et al., 2014). In a recent fMRI study using a graph structure of pictures, the adaptation signals in the hippocampal-entorhinal cortex were suppressed for shorter distances on the graph, indicating that non-spatial relationships were also encoded in these regions (Garvert et al., 2017). Other neuroimaging studies have shown that activations in the left posterior hippocampus were enhanced during retrieval compared with the encoding of word pairs (Prince et al., 2005) and that better recollection of proverbs was associated with a larger volume of the bilateral posterior hippocampus (Poppenk and Moscovitch, 2011). The results of the present study are consistent with these previous findings, in that the scheduled appointments included various cues of spatial and structural information in the calendar, which were especially abundant when participants used paper notebooks. Moreover, the retrieval of such encoded information was explicitly required by our retrieval task and was shown to elicit activations in the bilateral posterior hippocampus.

Concerning activation in the visual cortex, a previous study reported that the visual cortex was activated during the retrieval of pictorial visual information without actual visual stimulation (Wheeler et al., 2000). The visual areas play a key role in visual imagery as well, and activations in those regions could be affected by focal attention during imagery (Sakai and Miyashita, 1994). Indeed, a study with fMRI decoding revealed activation in the V1–V3 when participants reported visual imagery of an object during dreaming, about which was inquired afterward (Horikawa et al., 2013). Another study reported that retrieval of visual information was related to activation patterns in the V1–V3, and further showed that the activation patterns in the hippocampus predicted the mnemonic strength (Bosch et al., 2014). As regards the precuneus, a positron emission tomography (PET) study with a paired-word retrieval task showed memory-related activation for both visual and auditory stimuli, indicating a modality-general role of the precuneus (Krause et al., 1999). The internal representation for visual imagery of the encoded calendar provides a plausible account for our results, in which the paper notebook provides richer information than mobile devices.

According to our previous study, the left F3op/F3t, right LPMC, and right F3op/F3t are included in the network for syntax and its supportive system (Network I; Kinno et al., 2014), whereas the left LPMC is critical to the network for syntax and input/output interface (Network II). In the present study, we observed activation in the left F3t/F3O, which is an essential part of the network for syntax and semantics (Network III). Thus all three networks that are crucial for syntactic processing were involved in the retrieval of scheduled appointments. The enhanced activations for the Note group suggest that the use of paper notebooks even influenced natural language processes, possibly reflecting the encoding of specific episodes.

Our present experiments demonstrated that brain activations related to memory, visual imagery, and language during the retrieval of specific information, as well as the deeper encoding of that information, were stronger in participants using a paper notebook than in those using electronic devices. Our results suggest that the use of a paper notebook affects these higher-order brain functions, and this could have important implications for education, particularly in terms of the pros and cons of e-learning. The expanded use of mobile devices or computers could undercut the use of traditional textbooks and paper notebooks, which may in fact provide richer information from the perspective of memory encoding. Further research is needed to elucidate the actual changes in brain activation due to the long-term exposure to mobile devices.

## Data Availability Statement

The raw data supporting the conclusions of this article will be made available by the authors, without undue reservation.

## Ethics Statement

The studies involving human participants were reviewed and approved by the Institutional Review Board of the University of Tokyo, Komaba Campus. The patients/participants provided their written informed consent to participate in this study.

## Author Contributions

KU and KLS designed the study, analyzed the data, and wrote the manuscript. TI and TY contributed to the initial discussion. KU conducted the experiment. All authors contributed to the article and approved the submitted version.

## Conflict of Interest

TI and TY were employed by the company NTT Data Institute of Management Consulting, Inc. The remaining authors declare that the research was conducted in the absence of any commercial or financial relationships that could be construed as a potential conflict of interest.

## Footnotes

^1^http://www.fil.ion.ucl.ac.uk/spm

^2^http://www.gin.cnrs.fr/AAL2/

^3^http://Neuromorphometrics.com/

^4^http://marsbar.sourceforge.net/
